# Straw-Mediated Restructure of Arbuscular Mycorrhizal Fungal Community by Selectively Shifting Edaphic Biogeochemistry in Tea Plantations of South Henan, China

**DOI:** 10.3390/jof12040271

**Published:** 2026-04-09

**Authors:** Xiangchao Cui, Dongmeng Xu, Jiaju Wang, Yu Zhang, Shuping Huang, Wei Wei, Ge Ma, Mengdi Li, Junhui Yan

**Affiliations:** 1 Henan Key Laboratory for Synergistic Prevention of Water and Soil Environmental Pollution, School of Geographic Sciences, Xinyang Normal University, Xinyang 464000, China; dongmengxu1221@gmail.com (D.X.); 19139679891@163.com (J.W.); huangshuping8808@126.com (S.H.); weiweiqyz@xynu.edu.cn (W.W.); mage@xynu.edu.cn (G.M.); lmd20210605@163.com (M.L.); yanjh2015@126.com (J.Y.); 2 College of Plant Protection, Shandong Agricultural University, Tai’an 271001, China; yuzhang@sdau.edu.cn; 3 North-South Transitional Zone Typical Vegetation Phenology Observation and Research Station of Henan Province, Xinyang Normal University, Xinyang 464000, China

**Keywords:** straw application (SP), mycorrhizal colonization (MC), community structure, co-occurrence network, metal ions

## Abstract

Background: Straw application (SP) is an important agronomic practice in sustainable agriculture, yet its effects on arbuscular mycorrhizal (AM) fungal communities in tea plantation soils remain poorly understood. Methods: This study investigated the responses of AM fungi to SP in tea plantations in south Henan, China, by assessing colonization characteristics, community composition, diversity, co-occurrence networks, and soil environmental drivers. Results: SP significantly increased the mycorrhizal colonization rate (MC), by 59.4%. High-throughput sequencing (26,865 sequences and 406 ASVs) revealed that SP reduced the dominance of *Claroideoglomus* (32.2% to 10.5%) and *Glomus* (51.01% to 46.7%) while enriching *Paraglomus* and *Acaulospora*. Although the α-diversity was unaffected, the β-diversity significantly shifted, indicating community homogenization under SP. Differential taxa analysis confirmed genus-specific responses, and co-occurrence networks showed a simplified topology (nodes: −18.4%; edges: −33.4%) but maintained stability, with increased module specialization (*Z_i_* and *P_i_*). Soil properties explained 80.0% of the variation in AM fungal parameters, with pH and available phosphorus (AP) as key drivers. SP shifted environmental filters from nitrogen/carbon-related factors to metal ions (Al^3+^ and Ca^2+^), altering geochemical conditions. Conclusions: SP selectively reshapes AM fungal communities by altering soil microenvironments and selectively modulating the AM fungal community while maintaining network stability. This study provides new insights into the microbial mechanisms of SP and a basis for sustainable, AMF-based tea plantation management.

## 1. Introduction

Tea (*Camellia sinensis* (L.) Kuntze), a member of Theaceae, is a globally significant perennial cash crop, cultivated in approximately 50 countries, with a total area of 5.54 million hectares, including China, according to the data of the Food and Agriculture Organization of the United Nations in 2024 [1]. However, intensive, long-term monoculture in tea plantations often induces soil degradation, manifesting as acidification, nutrient imbalance, and a decline in microbial diversity and function [2]. The adoption of sustainable soil management practices has gained momentum to counteract these detrimental effects. Among these, the application of organic amendments—such as crop straw—is a widespread strategy to enhance soil organic matter (SOM), improve soil structure and moisture retention, and promote nutrient cycling [3,4]. It is also reported that straw application (SP) can increase crop yield by reducing exchangeable Al^3+^ by increasing pH and shifting the proportion of amorphous to free Fe/Al_ox_ in the acidic soils, enhance SOC storage and stability by promoting organo-Ca associations [5,6], and modulate the composition and activity of key functional microbial groups involved in these biogeochemical processes [7].

Arbuscular mycorrhizal (AM) fungi, among the well-known functional microbes, are key functional soil microbes that form symbiotic associations with most terrestrial plants, including tea trees [8,9]. AM fungi play vital roles in enhancing host phosphorus (P) uptake [10], improving soil aggregation and water retention, increasing plant tolerance to abiotic and biotic stresses [11,12,13], e.g., mitigating Al toxicity [14], and potentially influencing crop yield and quality [15]. In tea plants, AM fungi have been shown to promote plant growth and improve tea quality [16] by upregulating the expression of relevant genes, especially under drought stress [17]. The results of a long-term soil fertility gradient experiment showed that AM fungal assembly dynamics can enhance maize productivity [18], and agronomic management can increase the complexity of the AM fungal network to sustain wheat production [19]. While numerous pot experiments have demonstrated the benefits of AM fungal inoculation on tea plant yield, quality, and stress resistance, systematic research on the natural AM fungal communities within tea plantation ecosystems remains limited. The composition and function of AM fungal communities in other ecosystems are often affected by edaphic factors such as pH, nutrient availability, SOM, and moisture [20,21,22,23,24]. In the microbial ecosystem, the assembly of AM fungal communities is governed by deterministic processes such as environmental filtering, where abiotic conditions select (e.g., soil pH and nutrient availability) [7,20] for taxa with suitable traits, and biotic interactions, e.g., competition [25], which together shape community structure. Environmental filtering plays a pivotal role in determining AM fungal community assembly and distribution patterns [26]. These factors are often directly altered by the addition of organic amendments such as crop straw [27]. Existing studies have shown that SP can influence AM fungal diversity and colonization in various cropping systems, largely through modifications in soil physicochemical properties and carbon dynamics [28,29,30]. However, in the specific context of intensively managed, long-term monoculture tea plantations—characterized by inherently acidic soils with Al toxicity—the responses of AM fungi to SP remain poorly understood. In particular, there is limited knowledge regarding how SP reshapes AM fungal root colonization characteristics, community assembly, taxa interactions within a species or genus, and the associated topological keystones, as well as the underlying environmental drivers in tea plantation ecosystems.

Therefore, this study was conducted in the tea production area of South Henan Province, China, a renowned region for premium tea, Xinyang Maojian. We aimed to investigate the impact of SP on AM fungi in tea plantation soils. Specifically, we assessed (i) AM fungal colonization characteristics, including spore density (SD), hyphal length (Hypha), mycorrhizal colonization rate (MC), and glomalin-related soil protein content (GRSP); (ii) community diversity and composition via high-throughput sequencing (HTS); (iii) co-occurrence network topology and stability; and (iv) the key soil physicochemical factors driving these changes. We hypothesized that SP acts as a significant environmental filter, selectively altering AM fungal community structure and taxa interactions, with a consequent shift in the dominant edaphic drivers. This research provides novel insights into the microbial mechanisms underlying straw-mediated soil improvement and offers a scientific basis for developing sustainable, AM fungi-informed management practices in tea plantation ecosystems.

## 2. Materials and Methods

### 2.1. Description of Sites and Sampling

**Figure 1:**
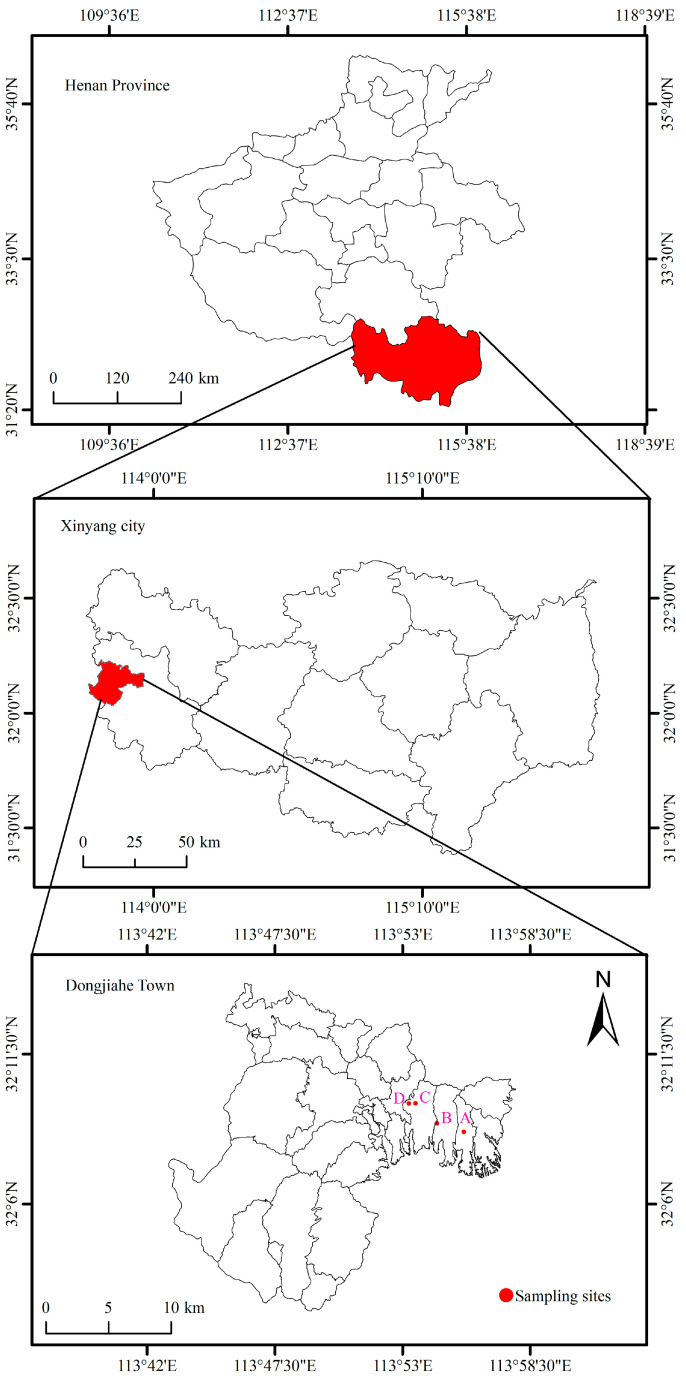
The geographic location of the study area around the tea plantations in Dongjiahe Town of Xinyang City, China. A, soil sampling sites in tea plantation A; B, soil sampling sites in tea plantation B; C, soil sampling sites in tea plantation C; and D, soil sampling sites in tea plantation D.

This study was conducted in Dongjiahe Town (32°02′21″–32°23′23″ N, 113°45′28″–114°13′09″ E), Shihe District, Xinyang City, Henan Province, China, a primary production area for the renowned *Camellia sinensis*, Xinyang Maojian (Figure 1). This region is situated in a climatic transition zone where northern subtropical and warm temperate monsoon climates converge. It experiences marked seasonal contrasts in both temperature and precipitation patterns [31]. According to meteorological data from 2018, the area has an average annual temperature and precipitation of 16.6 °C and 992 mm, respectively, with the relative humidity averaging 74.7% (Table S1). The landscape consists of eroded hills and low mountains (100–800 m a.s.l.). The dominant yellow–brown soils (Haplic Luvisols per FAO) are light to sandy loams with a pH range of 4.7–6.5 [32]. Standardized fertilization includes a basal application (Oct–Nov) of calcium superphosphate (375–750 kg·ha^−1^) and potassium sulfate (225–375 kg·ha^−1^), supplemented with two ammonium sulfate topdressings (37–125 kg·ha^−1^ each) in February and post-spring harvest. After the spring tea harvest, the pruning operation usually takes place to remove older branches and leaves, allowing the tea plant to conserve nutrients and promoting the vigorous growth of fresh buds for the summer and autumn tea seasons.

Soil sampling was conducted on 25 April 2018, across four independent tea plantations (A, B, C, and D) within Dongjiahe Town (Figure 1) after spring tea harvest. This sampling period was selected due to the relatively stable precipitation conditions, which are representative of the predominant soil moisture regime throughout the year [33].

The experimental design involved two contrasting management practices: wheat straw mulching (WT) was applied in Plantations A and B, while rice straw mulching (RT) was applied in Plantations C and D (Table S2). The straw was maintained at a length of 15–20 cm. Two long-term treatments were established within each plantation: (1) the control (CK) without straw application for 10 years, and (2) the straw-amended (S) treatment receiving 15,000 kg·ha^−1^ of fresh matter annually for 10 years.

Sampling was conducted using a stratified random design to address spatial variability. Specifically, three 10 m × 10 m replicate plots were set up for each treatment in every plantation. Within each plot, after carefully removing the surface litter and the top 0–5 cm of soil to minimize recent environmental interference, five soil subsamples were collected diagonally from a 5–20 cm depth using a stainless-steel auger (5 cm diameter). Fine tea roots were collected along with the soil samples. The root samples were approximately 1 mm in diameter, and each root segment was 2–4 cm in length, with over 3 segments for each subsample. The five soil and root subsamples from the same plot were completely homogenized to form one composite sample, with each soil sample of approximately 1 kg fresh weight in a plastic ziplock bag, and each root sample in a plastic tissue-embedding cassette. These samples were immediately transported to the laboratory, the Henan Key Laboratory for Synergistic Prevention of Water and Soil Environmental Pollution at Xinyang Normal University (Xinyang, China), for analysis of soil physicochemical properties and subsequent molecular microbial sequencing. In total, 24 composite soil samples were obtained (4 plantations × 2 treatments × 3 replicates) for the following analysis. The composite soil samples were processed as follows: Approximately 300 g of fresh soil was stored at 4 °C to determine soil water content and analyze AM fungal parameters. Another aliquot (approximately 50 g) of fresh soil was stored at −80 °C for subsequent molecular biological analysis. The remaining soil was air-dried at room temperature after removing stones and root fragments with a 2 mm mesh sieve, and then used for routine physicochemical analyses.

### 2.2. AM Colonization Characteristics and Soil Physicochemical Property Analysis

MC was assessed according to Phillips and Hayman [34] with modifications. Fine roots in the embedding cassette were cleared in 10% KOH and stained with 0.05% trypan blue in lactic acid–glycerol. The stained roots were cut into 0.5–1 cm segments. Colonization was quantified using the gridline intersect method under a stereomicroscope, examining at least 100 root segments per sample.

SD in the soil was determined using the wet sieving and decanting method followed by sucrose centrifugation [35]. Briefly, 50 g of air-dried soil was suspended in water. The supernatant was passed through a series of sieves (20–60–200–400-mesh). The residues collected on the 400-mesh sieve were centrifuged in a 50% sucrose solution. Spores were collected and counted under a dissecting microscope, and the density was expressed as the number of spores per g of dry soil.

GRSP was extracted and quantified as described by Wright and Upadhyaya [36]. Specifically, easily extractable GRSP (EE-GRSP) was extracted from 1 soil with 8 mL of 20 mM citrate buffer (pH 7.0) at 121 °C for 30 min. The protein content in the supernatant was determined using the Bradford assay with bovine serum albumin as the standard.

Hyphae of AM fungi in the soil were measured using the membrane filter technique [37]. A 5 g air-dried soil sample was suspended in water with sodium hexametaphosphate as a dispersant. The suspension was passed through sieves (20–60–100–200-mesh). Hyphae collected on the 200-mesh sieve and the final filtrate on a 0.45 μm membrane filter were stained with trypan blue. AM fungal hyphae were identified by morphology (diameter > 2 μm, right-angle branching, and the presence of vesicles/arbuscules/spores) and quantified using the gridline intersect method at 200× magnification under a light microscope.

Soil physicochemical properties were determined as follows. Soil gravimetric water content (WC) was measured by drying fresh soil at 105 °C to a stable weight (−0.01~0.01 g) [38]. Soil pH and electrical conductivity (EC) were measured in a 1:2.5 (*w*/*v*) soil-deionized water (boiled) suspension and a 1:5 (*w*/*v*) suspension, respectively, after 30 min of equilibration [38]. Available phosphorus (AP) was extracted with Bray-I solution (0.03 M NH_4_F and 0.025 M HCl) and determined via molybdenum–antimony colorimetry [39]. SOM was quantified using the potassium dichromate oxidation method [40]. Inorganic nitrogen (NH_4_^+^-N and NO_3_^−^-N) was extracted with 2 M KCl and analyzed using a Continuous Flow Analytical System (Skalar San++, Delft, The Netherlands) [41]. Exchangeable Ca^2+^ and Al^3+^ were extracted via alkali fusion, and their concentrations were measured using inductively coupled plasma–atomic emission spectrometry (ICP-AES) [42]. The results of the soil properties are summarized in Table S3.

### 2.3. DNA Extraction and Sequencing

Each soil sample (0.5 g) was subjected to total genomic DNA extraction using the FastDNA SPIN Kit for Soil (MP Biomedicals, Irvine, CA, USA) as per instructions. DNA concentration and purity were determined using a NanoDrop ND-1000 spectrophotometer (Thermo Scientific, Waltham, MA, USA), and samples were subsequently stored at −20 °C before further use.

The 18S rRNA gene fragment specific to AM fungi was amplified using the primers AMV4.5NF (5′-AAGCTCGTAGTTGAATTTCG-3′) and AMDGR (5′-CCCAACTATCCCTATTAATCAT-3′) [43]. Each 50 μL polymerase chain reaction (PCR) mixture contained approximately 50 ng of genomic DNA, 2 μL of each primer (10 μM), 4 μL of dNTP mix (2.5 mM each), 5 μL of 10× PCR buffer, 0.4 μL of Taq DNA polymerase (2 U, TaKaRa, Kusatsu, Japan), and nuclease-free water to volume. Amplification was performed in a TP600 Thermal Cycler (TaKaRa, Japan) under the following conditions: initial denaturation at 95 °C for 5 min; 35 cycles at 95 °C for 45 s, 58 °C for 45 s, and 72 °C for 1 min; followed by a final extension at 72 °C for 7 min.

For each of the 24 soil DNA samples, triplicate PCRs were prepared, amplified, and subsequently pooled to minimize reaction-level bias. The pooled amplicons were purified using the QIAquick PCR Purification Kit (QIAGEN, Hilden, Germany) and quantified with a NanoDrop spectrophotometer (Thermo Scientific, Waltham, MA, USA). Equimolar amounts of the purified PCR products from all samples were combined to construct a sequencing library. Paired-end sequencing was subsequently conducted on an Illumina HiSeq Ten platform (Illumina, San Diego, CA, USA) in accordance with the standard protocols.

### 2.4. Data Analysis

An AM fungal community analysis was conducted on the 18S rRNA data generated by the Quantitative Insights into Microbial Ecology 2 (QIIME2,version 2024.10). Primer sequences corresponding to the 18S rRNA gene amplicons were removed using cutadapt [44], including the forward primer AMV4.5NF and the reverse primer AMDGR. Sequence quality was evaluated based on the Interactive Quality Plot, and trimming and filtering were performed accordingly. The sequences were then denoised, merged, and subjected to chimera removal using the DADA2 algorithm [45] to obtain amplicon sequence variants (ASVs). A rarefaction curve for the read numbers of AM fungi sequencing was generated to identify the sequencing depth, and the results (over 30,000 reads for each sample) are displayed in Figure S1. Taxonomic assignment of ASVs was performed using the MaarjAM database [46], and a phylogenetic tree was constructed for downstream analyses. For downstream analysis, QIIME 2 artifacts (.qza) were imported into R as phyloseq objects using the qiime2Rpackage (v0.99.20, https://github.com/jbisanz/qiime2R.git; accessed on 24 March 2025). All statistical analyses were conducted in R 4.5.0. Alpha diversity indices (Shannon, Richness, Pielou’s evenness, and Faith’s phylogenetic diversity (PD), the latter computed with the picante package) were calculated. The Shapiro–Wilk test was applied to assess the normality of alpha diversity indices [47]. Given the non-normal distribution, differences in alpha diversity were evaluated using the Wilcoxon rank-sum test. β diversity was visualized via principal coordinate analysis (PCoA) based on Bray–Curtis distances (vegan package), and its significance was tested with permutational multivariate analysis of variance (PERMANOVA) [48]. The relative abundance of dominant AM fungal taxa was compiled in Microsoft Excel 2021 based on ASVs data and visualized with OriginPro 2024b, and the significance of differences in community composition was assessed using the Mann–Whitney U test. The community composition results of the dominant AM fungi are displayed in Table S4.

Differential taxa analysis was performed using DESeq2 via the microeco package (version 1.14.0, accessed on 28 March 2025), which fits a generalized linear model (GLM) based on the negative binomial distribution. Following initial analysis with the Wald test, a Benjamini–Hochberg false discovery rate (FDR) correction was applied to adjust all *p*-values for multiple comparisons [49]. The biomarker information of AM fungi based on the log_2_ fold change (log_2_FC) threshold (log_2_FC > |1|; *p* < 0.05) is displayed in Table S5.

Co-occurrence networks were reconstructed using the Meinshausen–Bühlmann neighborhood selection (mb) method within SpiecEasi [50,51] and visualized in Gephi (0.10.1), with correlation matrices generated using the Hmisc package (version 5.2-0, https://hbiostat.org/R/Hmisc/; accessed on 20 October 2024). The significance of the network topology characteristics was tested using Student’s Wilcoxon, and the stability was evaluated via the Stability Approach to Regularization Selection (StARS), with 50 subsamplings applied to determine the optimal sparsity level. The identification of topological keystones was conducted with the ggClusterNet package. According to established *Zi*-*Pi* thresholds, keystone nodes were classified into three types: module hubs (*Zi* > 2.5, *Pi* < 0.62), connectors (*Zi* < 2.5, *Pi* > 0.62), and network hubs (*Zi* > 2.5, *Pi* > 0.62) [52,53]. The metric of community cohesion was calculated based on the ASV correlation matrix and the relative abundance of ASVs [54]. The within-/among-module connectivity results of AM fungal topological keystones are displayed in Table S6.

The Mantel test and environmental factor association analysis were performed using the linkET package (version 0.0.3) [55]. The identification and quantification of environmental drivers involved two sequential analyses. First, distance-based redundancy analysis (dbRDA) implemented in the vegan package [48] was used to identify significant factors. Next, the relative contribution of each driver was quantified via variance partitioning analysis using the rdacca.hp package [56]. Permutation tests were applied throughout to assess the statistical significance of all models. The permutation results on the effects of soil physiochemical properties on the tbRDA, β-diversity, and the topological keystones of AM fungi are displayed in Table S7. Linear mixed models (LMMs) were employed in SPSS 22.0 to analyze AM fungal parameters (colonization, α- and β-diversity, and community cohesion) [57]. This approach was chosen to control for the random variation among plantation groups (A, B, C, and D), which was fitted as a random effect. The fixed effects in the model were straw treatment (CK and S) and straw type (WT and RT), allowing us to test the main effect of straw application and its potential dependency on straw type. The results of this analysis are shown in Table S8.

## 3. Results

### 3.1. AM Fungal Colonization Characteristics

**Figure 2:**
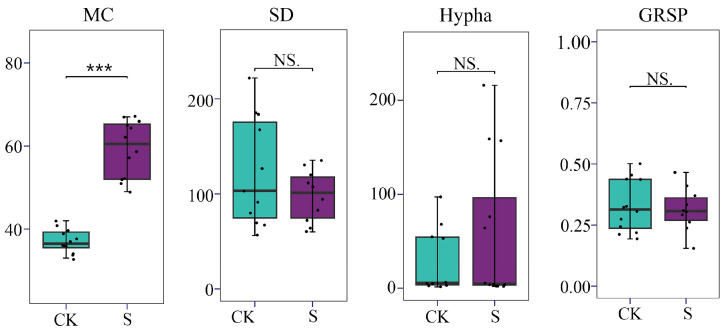
The comparisons of colonization characteristics of arbuscular mycorrhizal (AM) fungi in the tea plantation soils. CK, sample in the tea plantations without straw amendment; S, sample in the tea plantations with straw amendment. MC, mycorrhizal colonization; SD, spore density; Hypha, hyphal length; GRSP, glomalin-related soil protein. ***, significance with *p* < 0.001; NS, no significance with *p* > 0.05.

The AM fungal colonization characteristics were assessed by the indices of MC, SD, Hypha, and GRSP (Figure 2). Across all the samples, the rate of MC in the tea roots ranged from 33% to 66%, with SD values between 47 and 252 spores per gram of dry soil, Hypha between 2 and 216 cm per gram of dry soil, and a GRSP content ranging from 0.193 to 0.771 mg per gram of dry soil.

SP resulted in a significant increase in MC by 59.4% compared with the control group (CK) (*p* < 0.05), with the mean value rising from 38% under CK to 59% under SP. In contrast, no statistically significant differences were detected in SD, Hypha, and GRSP between the SP and CK (*p* > 0.05). These findings indicate that, among the parameters evaluated, MC is a well-responsive indicator to SP in tea plantation soils. The LMM results (Table S8a–c) showed that SP caused a significant difference in both MC and SD (*p* < 0.05). Moreover, tea plantations, as a random effect, did not affect the results of AM fungal colonization characteristics (*p* > 0.05), and straw type (RT and WT) also did not significantly influence the AM fungal indices (*p* > 0.05). This indicates that the straw type and tea plantation had a limited effect on AM fungal indices relative to the overall SP effect.

### 3.2. AM Fungal Community Composition

**Figure 3:**
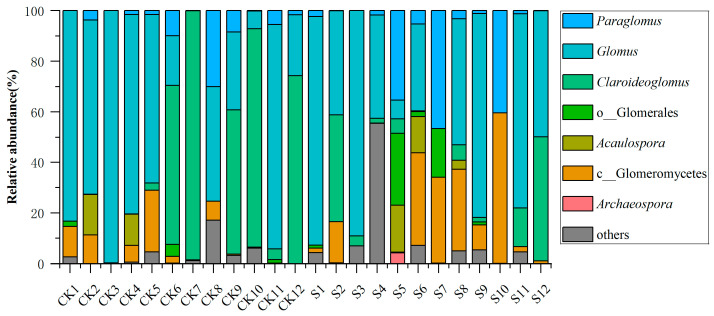
The relative abundance of arbuscular mycorrhizal (AM) fungi in different tea plantations with different straw treatments (CK and S). CK, sample in no-straw-amended tea plantations; S, sample in the straw-amended tea plantations.

There were 26,865 high-quality sequences obtained using HTS, which were clustered into 406 ASVs to assess the AM fungal community composition. The taxonomic analysis showed that *Claroideoglomus* and *Glomus* were the dominant genera across both CK and S treatments (Figure 3 and Table S4). Under the S treatment, the average relative abundance of *Claroideoglomus* and *Glomus* was, respectively, decreased from 32.2% to 10.5%, and from 51.01% to 46.7%. In contrast, the relative abundances of *Paraglomus*, *Acaulospora*, Glomeromycetes, and Glomerales exhibited increases, reaching 11.5%, 3.0%, 16.2%, and 4.3%, respectively. *Archaeospora* was exclusively detected in S. These results indicate that SP significantly altered the AM fungal community structure, favoring certain taxa such as *Paraglomus* and *Acaulospora*, while reducing the dominance of *Claroideoglomus* and *Glomus*.

### 3.3. α- and β-Diversity of AM Fungi

**Figure 4:**
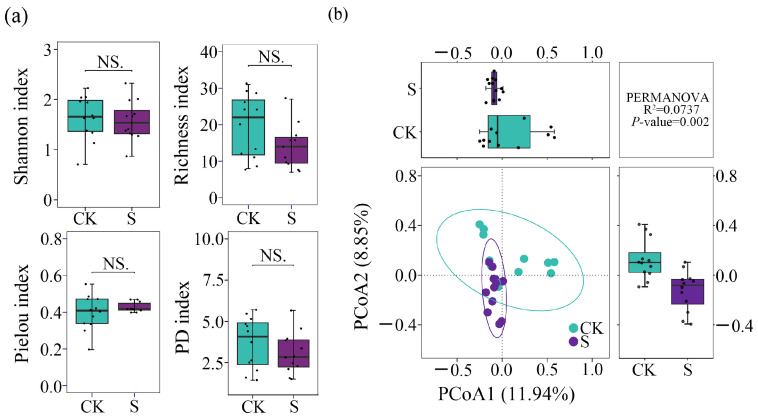
(**a**) The *α*-diversity index comparisons of Shannon, Richness, Pielou, and phylogenetic diversity (PD) of AM fungi (CK and S). (**b**) The principal coordinate analysis (PCoA) of AM fungi based on Bray–Curtis distances at the ASV level. CK, sample in no-straw-amended tea plantations; S, sample in the straw-amended tea plantations. PERMANOVA, permutational multivariate analysis of variance. NS, no significance with *p* > 0.05.

The α-diversity of AM fungi, as indicated by the Shannon, Richness, Pielou, and PD indices, showed no significant differences between CK and S (*p* < 0.05; Figure 4a). In contrast, β-diversity analysis based on Bray–Curtis distances at the ASV level revealed clear distinctions in fungal community structure between CK and S. PCoA showed that the first two coordinates collectively accounted for 20.79% of the total variance (Figure 4b). Although samples from CK and S treatments did not separate distinctly along PCoA1 or PCoA2, PERMANOVA analysis confirmed a significant difference in community composition between treatments (*R*^2^ = 0.0737, *F* = 1.75, and *p* = 0.002). Samples in the S group exhibited a tighter clustering pattern, suggesting that SP promoted greater homogeneity in the AM fungal community. The LMM results (Table S8a–c) showed that, except for the PCoA 2 of β-diversity, the α- and β-diversity displayed no significance between CK and S (*p* > 0.05). Tea plantations, as a random effect, did not affect the results of AM fungal colonization characteristics (*p* > 0.05). Straw type also did not show a significant effect on the treatment, indicating a limited effect of straw type and tea plantation on AM fungal diversity relative to the overall SP effect, while physicochemical properties differed.

### 3.4. Differential Taxa Analysis

**Figure 5:**
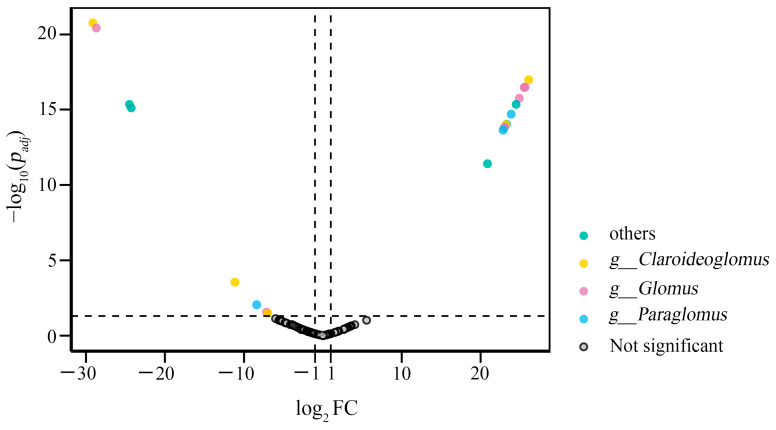
The differential taxa result of AM fungi between CK and S by the index of log_2_FC based on the AM fungal abundance. FC, fold change.

Differential taxa analysis was performed using DESeq2 to identify ASVs that were significantly affected by SP. A total of 20 ASVs exhibited significant differential abundance (|log_2_FC| > 1; *p* < 0.05), with 12 ASVs upregulated and eight downregulated in S compared with CK, which were confirmed as biomarkers (Figure 5). Key biomarkers included ASV4 (assigned to *Claroideoglomus*), which was significantly enriched in CK (log_2_FC = −29.1; *p* < 0.05), and ASV6 (also identified as *Claroideoglomus*), which was markedly upregulated in S (log_2_FC = 26.1; *p* < 0.05). At the genus level, *Glomus* and *Paraglomus* exhibited response trends similar to those of *Claroideoglomus*. For instance, ASV14 and ASV29 (both annotated as *Glomus*) were suppressed under S (*p* < 0.05), whereas ASV8 (*Glomus*) was significantly enriched in S (*p* < 0.05) (Table S5). These results suggest that SP induces genus-specific shifts in AM fungal taxa, highlighting the differential responsiveness within closely related groups.

### 3.5. Co-Occurrence Network Analysis of AM Fungi

**Figure 6:**
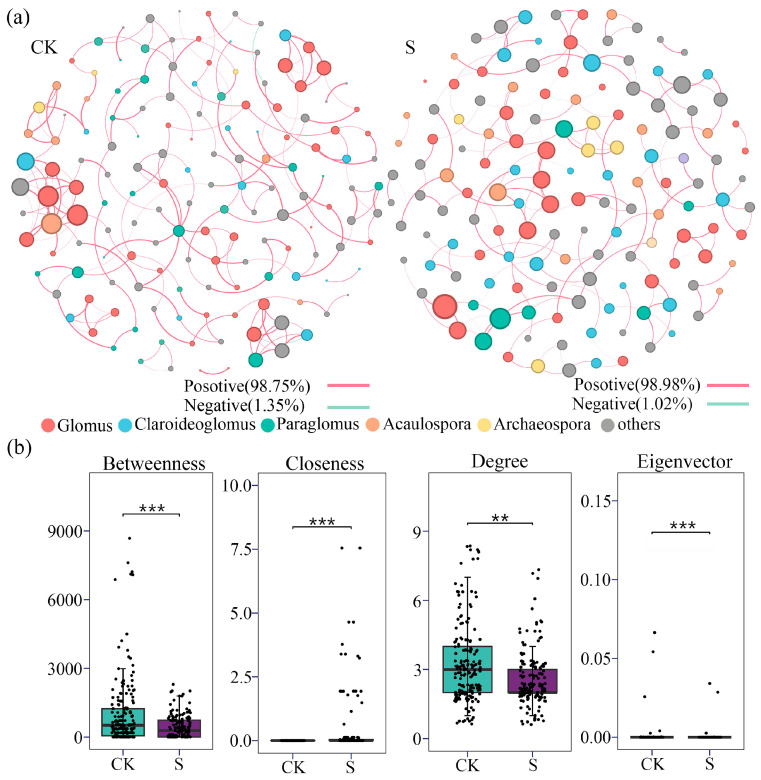
(**a**) The soil AM fungal co-occurrence network analysis of CK and S; (**b**) the topological parameters comparisons of co-occurrence networks of AM fungi between CK and S. CK, sample in no-straw-amended tea plantations; S, sample in straw-amended tea plantations. The network nodes with different colors mean different gena taxa of AM fungi. The edge with different colors indicates positive or negative correlation. Betweenness—betweenness centrality to measure a node’s role as a bridge in the network; closeness—closeness centrality to indicate a node’s overall proximity to all other nodes; degree—degree centrality to display the number of direct connections of a node; eigenvector—eigenvector centrality to reflect a node’s influence based on its connections’ importance. ***, significance with *p* < 0.001; **, significance with *p* > 0.01.

**Figure 7:**
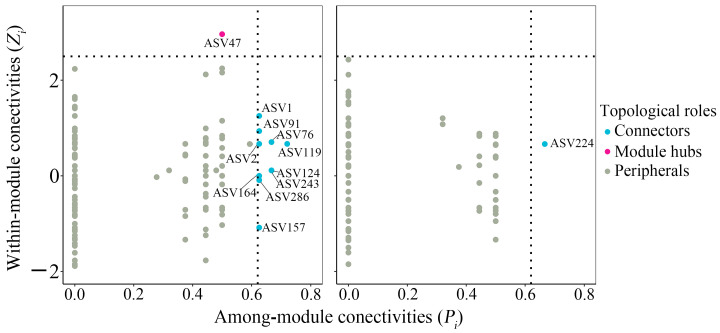
The within-/among-module connectivity results of the AM fungal co-occurrence network. CK, sample in no-straw-amended tea plantations; S, sample in straw-amended tea plantations. The gray nodes mean peripherals (*Z_i_* < 2.5; *P_i_* < 0.62); The red nodes indicate module hubs (*Z_i_* > 2.5; *P_i_* < 0.62). The blue nodes mean connectors (*Z_i_* < 2.5; *P_i_* > 0.62). The dotted line on the *P_i_* axis indicates *P_i_* = 0.62, and the dotted line on the *Z_i_* axis indicates *Z_i_* = 2.5.

The AM fungal co-occurrence network results are displayed in Table 1 and Table 2, and Figure 6 and Figure 7. Both the CK and S networks exhibited highly modular structures, characterized by high modularity indices (*Q_CK_* = 0.893 and *Q_S_* = 0.910) and clustering coefficients (*C_CK_* = 0.341 and *C_S_* = 0.332) (Table 1). The dominant taxa across both networks were *Glomus* (27.03% in CK; 29.14% in S), *Claroideoglomus* (11.89% in CK; 15.89% in S), and *Paraglomus* (9.73% in CK; 11.26% in S) (Table 2). SP significantly reduced network complexity, as reflected by an 18.4% decrease in the number of nodes, a 33.4% reduction in edges, and declines of 18.4% in average degree and 21.2% in network diameter. In addition, the proportion of negative correlations decreased by 23.1% (Table 1). At the node level, topological feature analysis revealed that SP significantly reduced betweenness centrality, degree centrality, and eigenvector centrality (*p* < 0.05), while increasing closeness centrality (*p* < 0.05) (Figure 6b). Positive interactions predominated in both the CK and S networks, accounting for 98.75% and 98.98% of total edges, respectively (Figure 6a), indicating that positive associations were prevalent among AM fungal taxa under both conditions.

Key species analysis further indicated a structural reorganization in the S network: the CK network contained 10 connectors and one module hub, primarily affiliated with *Claroideoglomus* (18.2%), *Glomus* (36.4%), and *Paraglomus* (27.3%) (Figure 7 and Table S6). In contrast, the S network retained only one connector, classified under Mucoromycota. Following SP, the average *Z_i_* value of topological keystones increased from 0.57 to 0.67, and the average *P_i_* value rose from 0.63 to 0.67 (Figure 7 and Table S5), suggesting weakened inter-module connectivity but enhanced functional roles for certain taxa.

**Figure 8:**
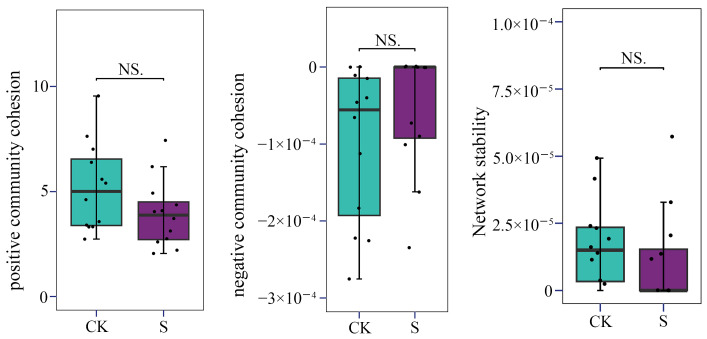
The boxplot comparison of positive and negative community cohesion and network stability of AM fungi. CK, sample in no-straw-amended tea plantations; S, sample in the straw-amended tea plantations. NS, no significance with *p* > 0.05.

The network-level structural cohesion of the AM fungal community was quantified based on the co-occurrence network matrix and relative abundance data (Figure 8). No significant differences were observed in either positive or negative cohesion between the CK and S treatments (*p* < 0.05). Thus, the AM fungal network stability of S also maintained the same level as that of CK. This indicates that although SP altered the topological structure and the topological keystone composition of the AM fungal community, the network stability of the fungal assemblage was maintained in tea plantation soils. Based on the LMM results (Table S8a–c), AM fungal network stability showed no significance between CK and S (*p* > 0.05), except for positive cohesion (*p* < 0.05). Tea plantation and straw type still had no significant effect on AM fungal network cohesion and stability, which means their limited effect relative to SP treatment.

### 3.6. Soil Factors Influencing AM Fungal Communities

**Figure 9:**
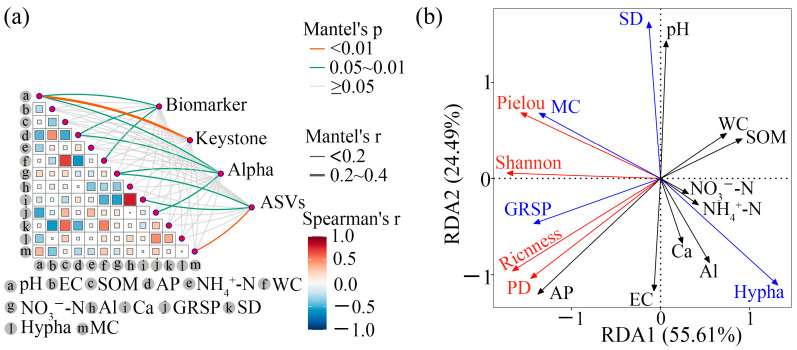
(**a**) The Mental’s tests of biomarker, keystone, alpha, and ASVs of AM fungi with soil properties. (**b**) The transformation-based redundancy analysis (tb-RDA) of AM fungal characteristics with soil properties. Biomarker, AM fungal biomarker species; keystone, AM fungal keystones; alpha, alpha diversity of AM fungal community; ASVs, AM fungal amplicon sequence variants. pH, soil pH; SOM, soil organic matter; EC, soil electrical conductivity; NH_4_^+^-N, ammonium nitrogen; AP, available phosphorus; WC, water content; NO_3_^−^-N, nitrate nitrogen; Al, aluminum; Ca, calcium; SD, AM fungal spore density; GRSP, glomalin-related soil protein; Hypha, AM fungal hypha length; MC, AM fungal mycorrhizal colonization. The black arrows represent soil physicochemical properties, the red arrows indicate the α-diversity of AM fungi, and the blue arrows denote AM fungal colonization indices.

Mantel tests revealed significant correlations between soil properties and AM fungal indicators (Biomarker, Keystone, α-diversity, and ASVs) in tea plantation soils (Figure 9a). Biomarkers were significantly associated with pH, AP, and WC (*p* < 0.05); keystone showed a significant correlation with pH (*p* < 0.05); α-diversity was significantly linked to pH, NO_3_^−^-N, and GRSP (*p* < 0.05); and ASVs were significantly influenced by NO_3_^−^-N and Ca^2+^ concentration. Furthermore, a significant correlation was observed between MC and ASVs, as well as between GRSP and α-diversity.

The tb-RDA based on AM fungal colonization indices and α-diversity indicated that soil physicochemical factors collectively explained 80.0% of the variation in these parameters (Figure 9b). Permutation tests and hierarchical partitioning (HP) further confirmed that pH was the primary factor affecting SD, while AP exerted the strongest influence on species richness, PD, and GRSP content (Table 3 and Table S7).

**Figure 10:**
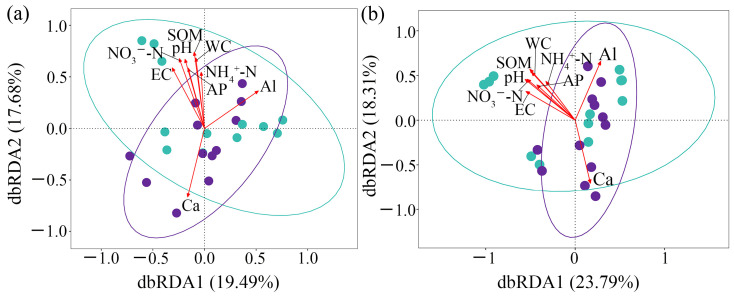
(**a**) The distance-based redundancy analysis (dbRDA) results of AM fungal *β*-diversity with the soil physicochemical characteristics; (**b**) the dbRDA results of the AM fungal differential and keystones with the soil physicochemical properties. CK, sample in no-straw-amended tea plantations; S, sample in the straw-amended tea plantations. pH, soil pH; SOM, soil organic matter; EC, soil electrical conductivity; WC, water content; AP, available phosphorus; NH_4_^+^-N, ammonium nitrogen; NO_3_^−^-N, nitrate nitrogen; Al, aluminum; Ca, calcium.

Distance-based redundancy analysis (dbRDA) of fungal β-diversity demonstrated that soil factors accounted for 37.2% of the total variance in AM fungal community structure (Figure 10a). HP identified pH, EC, SOM, NH_4_^+^-N, WC, NO_3_^−^-N, Al^3+^, and Ca^2+^ as the dominant drivers (Table 3). Permutation tests validated the significant effects of these factors (*p* < 0.05) (Table S6). The dbRDA results indicated that pH, EC, SOM, NH_4_^+^-N, WC, and NO_3_^−^-N were key determinants of community variation in CK, whereas Al^3+^ and Ca^2+^ were the primary drivers in S.

Analysis of differential and keystone species via dbRDA showed that soil physicochemical factors explained 42.1% of the variance (Figure 10b). HP again highlighted pH, EC, SOM, NH_4_^+^-N, WC, NO_3_^−^-N, Al^3+^, and Ca^2+^ as major contributing factors (Table 3). The significance of these variables was confirmed with permutation tests (*p* < 0.05) (Table S6). Consistent with the overall β-diversity results, pH, EC, SOM, NH_4_^+^-N, WC, and NO_3_^−^-N predominantly shaped community variation in CK, while Al^3+^ and Ca^2+^ played a more critical role in S. These findings underscore that SP alters the primary environmental filters, such as WC, pH, EC, and SOM, governing AM fungal assembly, shifting the dominant drivers from nitrogen- and carbon-related factors to metal ion dynamics.

## 4. Discussion

The association between Camellia sinensis and AM fungi is well-documented, and multiple species have been identified in tea soils, such as *Acaulospora laevis*, *Acaulospora gerdemannii*, *Glomus dolichosporum*, *Glomus clarum*, *Glomus etunicatum*, *Glomus geosporum*, and *Gigaspora tuberculata* [8,9]. Consistent with these reports, our findings that *Claroideoglomus* and *Glomus* were the dominant genera further confirm the formation of AM associations in tea plants. Our results demonstrate that SP significantly enhanced the MC of AM fungi by 59.4% in tea plantation soils, while SD, Hypha, and GRSP contents remained unaffected. This suggests that MC serves as a sensitive indicator for assessing the response of AM fungi to environmental changes and agricultural management practices [58,59,60], which directly reflects the rapid establishment and enhancement of the symbiotic relationship via SP between AM fungi and tea plants. The increase in MC likely reflects an enhanced carbon exchange between the host plant and the AM fungi [29,60], supported by the additional carbon source from straw decomposition, with AM fungi also promoting straw decomposition [60,61], which indicates that SP promotes AM colonization in tea plantations. In contrast, the lack of significant changes in SD, Hypha, and GRSP suggests that differential responses of the reproductive and structural components of the AM fungal community exist with the agricultural management or environmental factors changing [62]. It was reported that SP had no significant effect, or had a negative effect, on MC or richness in dryland farming systems [7,27,30], indicating that the response may be ecosystem-specific [23,63]. This discrepancy between MC and other indices underscores that different life-history traits of AM fungi (colonization vs. reproduction) exhibit distinct sensitivities to agricultural management practices. The tea plants likely allocated more photosynthates to the roots in response to the altered nutrient dynamics from straw decomposition [60], thereby favoring fungal colonization within the roots rather than immediate investment in external hyphal growth or spore production.

The read number of AM fungi was over 32,000, and the coverage was over 0.9999, which indicated a good sequencing depth and coverage. HTS analysis in this study revealed that SP significantly altered the AM fungal community structure in tea plantation soils. The dominant genera in CK, *Glomus*, and *Claroideoglomus* were consistent with previous observations in tea garden ecosystems [63]. Under S treatment, the relative abundance of *Claroideoglomus* decreased sharply from 32.2% to 10.5% [21], while *Glomus* declined moderately from 51.01% to 46.7%. In contrast, the relative abundances of *Paraglomus*, *Acaulospora*, Glomeromycetes, and Glomerales increased to 11.5%, 3.0%, 16.2%, and 4.3%, respectively. *Archaeospora* was exclusively detected in S. This compositional shift demonstrates that SP acts as an effective environmental filter based on the observed correlational patterns, selectively favoring certain taxa, such as *Paraglomus* and *Acaulospora*, while reducing the dominance of *Claroideoglomus* and *Glomus*. The reorganization was likely driven by alterations in the soil microenvironment induced by SP (Table S3). Specifically, genera such as *Glomus* are known to be sensitive to changes in soil nutrients and pH, whereas *Acaulospora* tends to be more adapted to acidic conditions [8,64,65]. As tea plantation soils are inherently acidic, straw decomposition could further modify soil pH and nutrient availability (e.g., forms of carbon, nitrogen, and phosphorus), collectively promoting community succession [21]. The enrichment of specific taxa and the appearance of *Archaeospora* in response to SP may be associated with the soil improvement [15] caused by straw. This potential link warrants further validation via targeted nutrient uptake experiments.

The analysis of AM fungal diversity revealed a nuanced response to SP. The absence of significant changes in α-diversity indices (Shannon, Richness, Pielou, and PD) indicates that while SP altered AM fungal community composition, it did not affect the within-community diversity of AM fungi in the tea plantation soils. This finding suggests an inherent stability in the overall diversity of the AM fungal community in these soils, which aligns with observations in other agricultural systems [66,67]. In contrast, the significant shift in β-diversity confirmed with PERMANOVA (*R*^2^ = 0.0737; *p* = 0.002) demonstrates that straw may act as a potential environmental filter, systematically restructuring the AM fungal community structure [7]. The low R^2^ value from PERMANOVA indicates that SP has limited explanatory power for AM fungal β-diversity, consistent with the multifactorial nature of AM fungal community composition [20,21,22]. Therefore, the statistical result should be interpreted with caution, avoiding overestimation of the independent effect of straw. In discussing its ecological significance, the findings should be validated by integrating other community metrics, such as symbiotic network analysis and changes in key taxa. The tighter clustering of samples from the straw-treated group in the PCoA plot (explaining 20.79% of the total variance) indicates a trend toward homogenization of the AM fungal community in straw-amended soils. This suggests that SP created a more uniform soil microenvironment—likely through standardized alterations in pH, carbon availability, and nutrient dynamics during its decomposition—which selectively favored a specific subset of AM fungal taxa, leading to a convergent community composition across different sampling sites and thereby reducing the heterogeneity observed in the control plots [3,68]. This pattern of management-driven compositional restructuring without immediate impacts on local α-diversity has also been observed in long-term fertilization studies [21]. Such shifts of the community toward a more uniform state under organic amendment may have implications for the functional stability of the soil ecosystem.

Differential taxa analysis revealed genus-specific responses of AM fungi to SP, highlighting significant functional divergence within phylogenetically related taxa. The contrasting patterns observed—specifically, the significant enrichment of *Claroideoglomus* ASV4 in CK (log_2_FC = –29.1) versus the marked increase in *Claroideoglomus* ASV6 in S (log_2_FC = 26.1)—demonstrate clear intrageneric niche partitioning. This phenomenon is likely driven by differential substrate preferences among closely related ASVs during straw decomposition, as organic inputs alter soil nutrient dynamics and resource availability [68]. Similarly, the divergent responses among *Glomus* ASVs (e.g., suppression of ASV14 and ASV29 versus enrichment of ASV8) align with existing reports of taxon-specific sensitivity to organic amendments [21]. These findings collectively suggest that functional traits, rather than phylogenetic affiliation alone, govern the adaptability of AM fungi to agricultural management practices such as SP [69].

Co-occurrence network analysis further elucidated how SP reshaped community architecture without compromising ecological stability. The reduction in network complexity—evidenced by decreases in node number (18.4%) and edge count (33.4%) and the disappearance of 10 connectors—reflects a streamlined community structure under straw-induced homogenization. This pattern is consistent with observations in cereal systems, where organic fertilization similarly simplifies microbial network topology [70,71]. Critically, the preservation of high modularity (Q > 0.89) and unchanged cohesion (*p* > 0.05) indicates that network stability was maintained despite structural reorganization, underscoring the resilience of AM fungal communities to management-induced disturbances, which was aligned with the research in extreme environments [22]. The increase in *Z_i_* and *P_i_* values suggests enhanced functional specialization within modules, a recognized adaptive strategy in amended soil environments [28]. Moreover, the predominance of positive correlations (>98%) in both networks confirms that positive interactions remain the cornerstone of AM fungal assembly, even when environmental filtering alters taxonomic composition. The observed restructured community of AM fungi with a good network stability indicates a broad tolerance range to the altered soil environment, which likely provides a resilient foundation for maintaining key symbiotic functions. However, the precise mechanistic links between these shifts in community composition and critical agronomic outcomes for tea plants—including P-uptake efficiency, yield, and quality parameters—remain unresolved and need to be studied further. Despite the nested design of our study, the linear mixed-effects model revealed consistent patterns. It indicated that the plantation group, incorporated as a random effect, did not exert a significant influence on AM fungal colonization, α- and β-diversity indices, or network cohesion and stability. Furthermore, the straw type, fitted as a fixed effect, showed only a limited effect, which was substantially weaker than the overall effect of SP.

The significant correlation observed between MC and ASVs, as well as between GRSP and α-diversity, indicates that the AM fungal community profile had the same response trend with AM colonization characteristics (MC and GRSP) to SP in the tea plantations. This suggests a potential link between the overall AM fungal community profile and colonization characteristics. Nevertheless, community profiles may not directly represent the fungi actively colonizing roots. Thus, future studies are needed to establish direct causal evidence. The multivariate analyses consistently demonstrated that soil physicochemical factors exerted differential but substantial influences on various aspects of the AM fungal community in the tea plantations. The collective explanation of 80.0% of the variation in colonization indices and α-diversity by soil factors underscores the profound role of the soil environment in shaping the symbiotic relationship and local diversity [72]. The identification of pH as the primary factor affecting the SD of AM fungi and its significant correlation with multiple AM fungal parameters aligns with established knowledge that soil pH is a master regulator of AM fungal physiology and distribution [73]. Similarly, the strong influence of AP on species richness and GRSP content can be attributed to the central role of AM fungi in plant phosphorus acquisition [20].

It is reported that SP fundamentally reconfigured the primary environmental filters, which governed AM fungal β-diversity [28,29,30]. The results of db-RDA and HP in this study were also consistent with these earlier findings. The shift in key drivers from nitrogen- and carbon-related factors (e.g., SOM, NH_4_^+^-N, and NO_3_^−^-N) in the control soils to metal ions (Al^3+^, Ca^2+^) in the straw-treated soils indicates that SP corresponds to a change in the relative importance of these environmental drivers. This alteration likely involves changes in soil pH, SOM, and cation exchange capacity, which, in turn, modulate the solubility, bioavailability, and potential toxicity of these metal ions [74,75,76]. This shift aligns with known functional traits of AM fungi. Certain taxa, such as *Acaulospora delicata* and *Gigaspora margarita*, exhibit considerable tolerance to Al toxicity [77], potentially through mechanisms such as upregulating proline biosynthesis in host plants to mitigate Al stress [14]. Concurrently, Ca^2+^ plays a dual role: it promotes AM fungal colonization [78] and is also central to the symbiotic signaling dialogue between the fungus and the host root [79]. This shift underscores the role of straw as both a nutrient source and an agent that modifies geochemical conditions, thereby applying a distinct selective pressure on the soil microbiome [80].

This study, through multi-faceted analyses, confirmed that SP significantly influences the colonization, community structure, network topology, and functional traits of AM fungi in tea plantation soils primarily by altering the soil microenvironment (e.g., pH, available nutrients, and metal ion dynamics) rather than merely acting as a nutrient source. This selects for more adaptive taxa and maintains community stability. These findings provide a theoretical basis for adopting SP as a management practice to enhance the sustainability of tea plantation ecosystems. Given that the investigation was centered on a key harvesting period, this study did not cover the dynamic responses of AM fungi to SP across different seasons or developmental stages of tea plants. Future research should focus on long-term monitoring to clarify the persistence of these effects [3] and integrate multi-omics [81] and other techniques to analyze the response mechanisms of AM fungal functional genes to straw input, thereby developing efficient mycorrhizal-straw synergistic management strategies tailored to different tea plantation ecosystems.

## 5. Conclusions

This investigation into AM fungal colonization and molecular biological indicators in tea plantation soils with and without SP revealed that SP significantly increased MC and reshaped AM fungal communities. Straw suppressed the relative abundance of dominant genera such as *Glomus* and *Claroideoglomus,* while promoting the enrichment of certain taxa such as *Paraglomus* and *Acaulospora*. Although no significant change was observed in the α-diversity of AM fungi, analyses of the β-diversity and differential taxa responses indicated that straw acted as an environmental filter, exerting a clear selective pressure on the AM fungal community. The co-occurrence network analysis revealed that SP simplified the network topology while maintaining the network-level structural stability of AM fungal communities. The RDA analysis demonstrated that SP significantly influenced AM fungal colonization, community structure, and interaction networks in the tea plantation soil by selectively altering the soil microenvironment, shifting the dominant drivers from carbon- and nitrogen-related factors (e.g., SOM, NH_4_^+^-N, and NO_3_^−^-N) to metal ions (Al^3+^ and Ca^2+^). Future studies should integrate measurements of phosphorus uptake, tea yield, and quality with controlled experiments manipulating Al/Ca availability to delineate the mechanistic pathway from straw amendment to plant performance.

## Figures and Tables

**Table 1:** The main topological parameters of the co-occurrence network of CK and S.

Parameters	CK	S	S/CK Ratio Variation
Node	185	151	−18.4%
Edge	296	197	−33.4%
Average degree	3.20	2.61	−18.4%
Network diameter	33	26	−21.2%
Graph density	0.017	0.017	0.0%
Modularity index (Q)	0.893	0.910	1.9%
Clustering coefficient (C)	0.341	0.332	−2.6%
Positive edges	0.987	0.990	0.3%
Negative edges	0.013	0.010	−23.1%

Node—a basic unit in the network, typically representing a microbial taxon; edge—a link between two nodes, representing a significant correlation; average degree—the average number of connections per node in the network; network diameter—the longest shortest path between any two nodes in the network; graph density—the ratio of actual edges to all possible edges in the network; modularity index (Q)—measures the strength of division of the network into modules; clustering coefficient (C)—measures the degree to which nodes tend to cluster together; positive edges—edges representing a positive correlation between nodes; negative edges—edges representing a negative correlation (e.g., mutual exclusion) between nodes. CK, sample in no-straw-amended tea plantations; S, sample in straw-amended tea plantations.

**Table 2:** The genus proportion of nodes in the co-occurrence network of CK and S.

Genus	CK	S
Others	49.73%	37.09%
*Glomus*	27.03%	29.14%
*Claroideoglomus*	11.89%	15.89%
*Paraglomus*	9.73%	11.26%
*Acaulospora*	1.62%	4.64%
*Archaeospora*	0%	1.99%

CK, sample in no-straw-amended tea plantations; S, sample in straw-amended tea plantations.

**Table 3:** The hierarchical partitioning (HP) results of the transformation-based redundancy analysis (tb-RDA) and the distance-based redundancy analysis (dbRDA) of AM fungi.

Group	Soil Physicochemical Properties	Unique Contribution	Average Share Contribution	Individual Contribution	Individual Percentage (%)
tbRDA of AM fungal α-diversity and colonization indices	pH	−0.0048	0.0179	0.0131	6.89
	EC	−0.0172	0.0522	0.0350	18.42
	SOM	−0.0487	0.0248	−0.0239	−12.58
	AP	0.0757	−0.0242	0.0515	27.11
	NH_4_^+^-N	−0.0032	−0.0030	−0.0062	−3.26
	WC	0.0431	−0.0468	−0.0037	−1.95
	NO_3_^−^-N	0.0197	−0.0156	0.0041	2.16
	Al	0.1727	−0.0715	0.1012	53.26
	Ca	0.0549	−0.0365	0.0184	9.68
db-RDA of AM fungal β-diversity	pH	0.0221	0.011		8.62
	EC	0.0343	0.0029	0.0372	9.69
	SOM	0.0437	0.0029	0.0466	12.14
	AP	0.0378	0.0012	0.039	10.16
	NH_4_^+^-N	0.0588	−0.0064	0.0524	13.65
	WC	0.0447	−0.002	0.0427	11.12
	NO_3_^−^-N	0.0384	0.0039	0.0423	11.02
	Al	0.0306	0.0149	0.0455	11.85
	Ca	0.0361	0.0093	0.0454	11.82
db-RDA of biomarker and keystone	pH	0.0221	0.0110	0.0331	8.62
	EC	0.0343	0.0029	0.0372	9.69
	SOM	0.0437	0.0029	0.0466	12.14
	AP	0.0378	0.0012	0.0390	10.16
	NH_4_^+^-N	0.0588	−0.0064	0.0524	13.65
	WC	0.0447	−0.0020	0.0427	11.12
	NO_3_^−^-N	0.0384	0.0039	0.0423	11.02
	Al	0.0306	0.0149	0.0455	11.85
	Ca	0.0361	0.0093	0.0454	11.82

pH, soil pH; EC, soil electrical conductivity; SOM, soil organic matter; AP, available phosphorus; NH_4_^+^-N, ammonium nitrogen; WC, water content; NO_3_^−^-N, nitrate nitrogen; Al, aluminum; Ca, calcium.

## Data Availability

The raw AM fungal sequencing data have been deposited in the Genome Sequence Archive (GSA) at the National Genomics Data Center (NGDC), the China National Center for Bioinformation/Beijing Institute of Genomics, the Chinese Academy of Sciences, under accession number CRA038522 (https://download.cncb.ac.cn/gsa6/CRA038522/, accessed on 14 February 2026); other data are contained within this article.

## Supplementary Materials

The following supporting information can be downloaded at https://www.mdpi.com/article/10.3390/jof12040271/s1, Figure S1: Rarefaction of AM fungi sequencing reads; Table S1: The 2017–2018 meteorological data of Xinyang; Table S2: Straw characteristics in the tea plantations; Table S3: Soil characteristics in different tea plantations; Table S4: The AM fungal dominant community composition; Table S5: The biomarker results based on the log_2_ fold change (log_2_FC) threshold; Table S6: The within/between-module connectivity results of the AM fungal topological keystones; Table S7: The permutation test of the effects of soil physiochemical characteristics on the tbRDA, β-diversity and the topological keystones of AM fungi. Table S8: (a) The type III tests of fixed effects in this study; (b): The estimates of fixed effects in this study; (c): The estimates of covariance parameters about the random effects in this study.

## Abbreviations

The following abbreviations were used in this manuscript:

AP	Available phosphorus
ASVs	Amplicon sequence variants
CK	Soil sample from the tea plantations without straw application
dbRDA	Distance-based redundancy analysis
EC	Electrical conductivity
FDR	False discovery rate
GLM	Generalized linear model
GRSP	Glomalin-related soil protein
HP	Hierarchical partitioning
LMM	Linear mixed model
MC	Mycorrhizal fungi
PCoA	Principal coordinate analysis
PD	Phylogenetic diversity
PERMANOVA	Permutational multivariate analysis of variance
*P_i_*	Among-module connectivity
Q	Modularity index
QIIME2	Quantitative Insights into Microbial Ecology 2
S	Soil sample from the tea plantations with straw application
SD	Spore density
SOM	Soil organic matter
SP	Straw application
tbRDA	Transformation-based redundancy analysis
WC	Water content
*Z_i_*	Within-module connectivity
