# Supplementary material for: Straw-Mediated Restructure of Arbuscular Mycorrhizal Fungal Community by Selectively Shifting Edaphic Biogeochemistry in Tea Plantations of South Henan, China
Source: J Fungi (Basel). 2026 Apr 9;12(4):271. doi: 10.3390/jof12040271 (PMC13117275; doi:10.3390/jof12040271)
Supplement: Supplementary file 1 [file jof-12-00271-s001.zip › Table S1.pdf]

Table S1 The 2017-2018 meteorological data of Xinyang

Year	Month	Mean monthly temperature (℃)	Mean monthly precipitation (mm)	Relative humidity averages (%)
2017	1	4.8	60.9	69.9
	2	6.9	41.1	65.0
	3	10.9	47.7	63.9
	4	18.2	83.0	62.6
	5	23.1	76.4	62.4
	6	25.3	94.0	72.3
	7	29.1	260.5	74.7
	8	27.4	218.1	81.2
	9	22.1	238.9	86.5
	10	15.2	215.9	86.8
	11	11.4	14.0	67.2
	12	6.1	5.4	57.6
2018	1	0.5	99.2	78.1
	2	5.6	25.9	63.7
	3	12.9	91.1	72.5
	4	18.4	63.2	67.5
	5	22.4	202.0	78.2
	6	26.7	65.4	72.4
	7	28.6	176.8	84.0
	8	28.1	106.7	85.7
	9	23.0	31.3	78.4
	10	17.7	5.1	61.1
	11	11.2	79.5	78.2
	12	4.0	45.8	76.4

Year	Mean annual temperature (℃)	Mean annual precipitation (mm)	Summer averages temperature (℃)	Winter averages temperature (℃)	Relative humidity averages (%)
2017	16.7	992.0	27.3	5.9	70.9
2018	16.6	1355.9	27.8	3.4	74.7
