# Supplementary material for: Straw-Mediated Restructure of Arbuscular Mycorrhizal Fungal Community by Selectively Shifting Edaphic Biogeochemistry in Tea Plantations of South Henan, China
Source: J Fungi (Basel). 2026 Apr 9;12(4):271. doi: 10.3390/jof12040271 (PMC13117275; doi:10.3390/jof12040271)
Supplement: Supplementary file 1 [file jof-12-00271-s001.zip › Table S2.pdf]

Table S2 Straw characteristics in the tea plantations

Straw types	N g/kg	P_2O_5 g/kg	Cellulose (%)	Hemicellulose (%)	Lignin (%)
Wheat	5.0~6.7	2.0~3.4	37.0~41.0	26.0~31.0	13.0~15.0
Rice	6.3~8.5	1.1~2.9	31.0~45.0	23.0~28.0	12.0~14.0
