# Supplementary material for: Straw-Mediated Restructure of Arbuscular Mycorrhizal Fungal Community by Selectively Shifting Edaphic Biogeochemistry in Tea Plantations of South Henan, China
Source: J Fungi (Basel). 2026 Apr 9;12(4):271. doi: 10.3390/jof12040271 (PMC13117275; doi:10.3390/jof12040271)
Supplement: Supplementary file 1 [file jof-12-00271-s001.zip › Table S3.pdf]

Table S3 Soil characteristics in different tea plantations

Treatment	pH	EC ($ms\cdot cm^{-1}$)	SOM ($g\cdot kg^{-1}$)	AP ($mg\cdot kg^{-1}$)	NH_4^--N content($mg\cdot kg^{-1}$)	NO_3^--N content($mg\cdot kg^{-1}$)	WC %	Al content ($mg\cdot kg^{-1}$)	Ca content ($mg\cdot kg^{-1}$)
CK1	6.25	143.3	22	9.56	15.1	1.35	19.62	10037	2192
CK2	6.4	154.2	20.1	8.37	16.9	1.88	23.46	1023	13
CK3	6.35	135.5	26.1	7.41	16.3	2.15	23.15	4258	615
CK4	5.56	114.2	31.9	8.37	15.7	1.46	28.87	9457	925
CK5	5.81	102.2	27.5	7.65	14.5	1.61	30.55	11675	1121
CK6	5.6	121.4	27.9	7.65	19.5	0.84	33.33	9430	1904
CK7	5.48	119.5	27.9	7.89	23.7	1.3	36.24	8351	972
CK8	5.98	158.1	21.7	14.34	8.3	1.31	22.55	15274	4252
CK9	5.61	138.1	18.1	12.19	16.6	0.87	23.15	8427	1036
CK10	5.32	217	20.3	12.43	26.9	1.31	20.77	12548	2488
CK11	6.04	94.2	25.5	9.08	18.8	1.24	19.9	15310	4143
CK12	5.86	103.1	24.9	9.56	21	1.41	24.69	5826	667
S1	6.36	55.1	24.4	8.13	15.9	1.61	25	6254	889
S2	6.13	148	25.9	8.60	15	1.21	27.88	10615	687
S3	6.11	133	22.8	8.13	17	0.84	23.76	10453	974
S4	6	188.9	25.3	8.37	15.3	1.44	26.58	9378	609
S5	6.05	91.6	29.7	7.41	7.4	1.44	40.45	11434	680
S6	6.3	97.4	24.6	7.17	16.6	1.42	34.41	12459	878
S7	6.45	101.7	26.3	7.17	17.6	1.14	37.74	9439	1408
S8	5.34	102.9	23.4	23.42	25.7	0.79	19.62	10857	2017
S9	6.03	161.7	24.2	8.37	16.2	0.88	23.46	14939	4373
S10	6.28	78.2	25.2	8.37	13.2	0.47	26.26	14347	3480
S11	6.14	133.2	25.1	7.89	14.7	1.55	22.85	17303	4727

S12	6.17	76.3	22.6	7.17	14.2	0.44	20.48	18790	5248

Electrical conductivity (EC), Soil organic matter (SOM), Available phosphorus content (AP), Soil gravimetric water content (WC).
