# Supplementary material for: Straw-Mediated Restructure of Arbuscular Mycorrhizal Fungal Community by Selectively Shifting Edaphic Biogeochemistry in Tea Plantations of South Henan, China
Source: J Fungi (Basel). 2026 Apr 9;12(4):271. doi: 10.3390/jof12040271 (PMC13117275; doi:10.3390/jof12040271)
Supplement: Supplementary file 1 [file jof-12-00271-s001.zip › Table S4.pdf]

Table S4 The AM fungal dominant community composition

Taxa level	Taxonomy	CK (%)	S (%)
Genes	Claroideoglomus	0.01~86.41	0.01~49.05
	Glomus	0~99.66	0~90.28
	c__Glomeromycetes	0~24.4	0.06~59.54
	Paraglomus	0~30.07	0~46.54
	Acaulospora	0~16.02	0~18.47
	o__Glomerales	0~4.82	0~28.47
	Archaeospora	0~0	0~4.17
	others	0~17.08	0~55.5
