# Supplementary material for: Straw-Mediated Restructure of Arbuscular Mycorrhizal Fungal Community by Selectively Shifting Edaphic Biogeochemistry in Tea Plantations of South Henan, China
Source: J Fungi (Basel). 2026 Apr 9;12(4):271. doi: 10.3390/jof12040271 (PMC13117275; doi:10.3390/jof12040271)
Supplement: Supplementary file 1 [file jof-12-00271-s001.zip › Table S5.pdf]

Table S5. The biomarker results based on the log2 Fold Chang (log2 FC) threshold

Regulation	Taxa	ASV	log2 FC	*Padj*
Down	k__Fungi, p__Mucoromycota, c__Glomeromycetes, o__Glomerales, f__Claroideoglomeraceae, g__Claroideoglomus	ASV4	-29.13	4.56E-23
	k__Fungi, p__Mucoromycota, c__Glomeromycetes, o__Glomerales, f__Glomeraceae, g__Glomus	ASV8	-28.69	2.02E-22
	k__Fungi, p__Mucoromycota, c__Glomeromycetes	ASV43	-24.49	9.80E-17
	k__Fungi, p__Mucoromycota, c__Glomeromycetes,	ASV134	-24.26	1.89E-16
	k__Fungi, p__Mucoromycota, c__Glomeromycetes, o__Glomerales, f__Claroideoglomeraceae, g__Claroideoglomus	ASV1	-11.15	0.000129904
	k__Fungi, p__Mucoromycota, c__Paraglomeromycetes, o__Paraglomerales, f__Paraglomeraceae, g__Paraglomus	ASV143	-8.40	0.004295778
	k__Fungi, p__Mucoromycota, c__Glomeromycetes, o__Glomerales, f__Glomeraceae, g__Glomus	ASV5	-7.14	0.014024463
	k__Fungi, p__Mucoromycota, c__Glomeromycetes, o__Glomerales, f__Claroideoglomeraceae, g__Claroideoglomus	ASV180	-7.00	0.017468365
Up	k__Fungi, p__Mucoromycota, c__Glomeromycetes, o__Glomerales, f__Claroideoglomeraceae, g__Claroideoglomus	ASV6	26.09	1.01E-17
	k__Fungi, p__Mucoromycota, c__Glomeromycetes, o__Glomerales, f__Glomeraceae, g__Glomus	ASV79	25.63	3.04E-17
	k__Fungi, p__Mucoromycota, c__Glomeromycetes, o__Glomerales, f__Glomeraceae, g__Glomus	ASV14	25.52	3.32E-17
	k__Fungi, p__Mucoromycota, c__Glomeromycetes, o__Glomerales, f__Glomeraceae, g__Glomus	ASV31	24.89	1.75E-16
	k__Fungi, p__Mucoromycota, c__Glomeromycetes, o__Glomerales	ASV11	24.50	4.53E-16
	k__Fungi, p__Mucoromycota, c__Paraglomeromycetes, o__Paraglomerales, f__Paraglomeraceae, g__Paraglomus	ASV111	23.87	2.02E-15

Regulation	Taxa	ASV	log2 FC	*Padj*
	k__Fungi, p__Mucoromycota	ASV30	23.28	9.47E-15
	k__Fungi, p__Mucoromycota, c__Glomeromycetes, o__Glomerales, f__Claroideoglomeraceae, g__Claroideoglomus	ASV9	23.21	1.01E-14
	k__Fungi, p__Mucoromycota, c__Paraglomeromycetes, o__Paraglomerales, f__Paraglomeraceae, g__Paraglomus	ASV84	23.03	1.53E-14
	k__Fungi, p__Mucoromycota, c__Glomeromycetes, o__Glomerales, f__Glomeraceae, g__Glomus	ASV29	23.02	1.53E-14
	k__Fungi, p__Mucoromycota, c__Paraglomeromycetes, o__Paraglomerales, f__Paraglomeraceae, g__Paraglomus	ASV112	22.84	2.29E-14
	k__Fungi, p__Mucoromycota	ASV312	20.87	3.92E-12
