# Supplementary material for: Straw-Mediated Restructure of Arbuscular Mycorrhizal Fungal Community by Selectively Shifting Edaphic Biogeochemistry in Tea Plantations of South Henan, China
Source: J Fungi (Basel). 2026 Apr 9;12(4):271. doi: 10.3390/jof12040271 (PMC13117275; doi:10.3390/jof12040271)
Supplement: Supplementary file 1 [file jof-12-00271-s001.zip › Table S6.pdf]

Table S6. The within/between-Module connectivity results of the AM fungal topological keystones

Treatment	Sequence	Taxa	Z_i	P_i	Roles
CK	ASV1	k__Fungi, p__Mucoromycota, c__Glomeromycetes, o__Glomerales, f__Claroideoglomeraceae, g__Claroideoglomus	1.26	0.63	Connectors
	ASV119	k__Fungi, p__Mucoromycota, c__Glomeromycetes, o__Glomerales, f__Glomeraceae, g__Glomus	0.67	0.72	Connectors
	ASV124	k__Fungi, p__Mucoromycota, c__Glomeromycetes, o__Glomerales, f__Claroideoglomeraceae, g__Claroideoglomus	0.11	0.67	Connectors
	ASV157	k__Fungi, p__Mucoromycota, c__Paraglomeromycetes, o__Paraglomerales, f__Paraglomeraceae, g__Paraglomus	-1.08	0.63	Connectors
	ASV164	k__Fungi, p__Mucoromycota, c__Paraglomeromycetes, o__Paraglomerales, f__Paraglomeraceae, g__Paraglomus	0.00	0.63	Connectors
	ASV2	k__Fungi, p__Mucoromycota, c__Glomeromycetes, o__Glomerales, f__Glomeraceae, g__Glomus	0.67	0.63	Connectors
	ASV243	k__Fungi, p__Mucoromycota	0.11	0.67	Connectors
	ASV286	k__Fungi, p__Mucoromycota, c__Glomeromycetes	-0.09	0.63	Connectors
	ASV47	k__Fungi, p__Mucoromycota, c__Glomeromycetes, o__Glomerales, f__Glomeraceae, g__Glomus	2.97	0.50	Module hubs
	ASV76	k__Fungi, p__Mucoromycota, c__Glomeromycetes, o__Glomerales, f__Glomeraceae, g__Glomus	0.71	0.67	Connectors
	ASV91	k__Fungi, p__Mucoromycota, c__Paraglomeromycetes, o__Paraglomerales, f__Paraglomeraceae, g__Paraglomus	0.94	0.63	Connectors

**Treatment**	**Sequence**	**Taxa**	Z_i	P_i	**Roles**
S	ASV224	k__Fungi, p__Mucoromycota	0.67	0.67	Connectors
