# Supplementary material for: Straw-Mediated Restructure of Arbuscular Mycorrhizal Fungal Community by Selectively Shifting Edaphic Biogeochemistry in Tea Plantations of South Henan, China
Source: J Fungi (Basel). 2026 Apr 9;12(4):271. doi: 10.3390/jof12040271 (PMC13117275; doi:10.3390/jof12040271)
Supplement: Supplementary file 1 [file jof-12-00271-s001.zip › Table S7.pdf]

Table S7. The permutation test of the effects of soil physiochemical characteristics on the tbRDA, β-diversity and the topological keystones of AM fungi

Group	Soil physicochemical characteristics	R^2	P	P_{adj}
tbRDA	pH	0.38	0.009	0.041
	EC	0.254	0.047	0.141
	SOM	0.147	0.188	0.338
	AP	0.547	0.001	0.009
	NH3-N	0.033	0.694	0.781
	WC	0.119	0.265	0.398
	NO3-N	0.016	0.839	0.839
	Al	0.165	0.13	0.293
	Ca	0.087	0.395	0.508
β-diversity	pH	0.419	0.003	0.007
	EC	0.373	0.014	0.018
	SOM	0.488	0.001	0.007
	AP	0.317	0.026	0.029
	NH3-N	0.263	0.045	0.045
	WC	0.415	0.004	0.007
	NO3-N	0.443	0.003	0.007
	Al	0.357	0.008	0.012
	Ca	0.425	0.004	0.007
topological keystone and Biomarker	pH	0.406	0.003	0.007
	EC	0.323	0.016	0.021
	SOM	0.483	0.002	0.006
	AP	0.278	0.039	0.041
	NH3.N	0.248	0.041	0.041
	WC	0.435	0.002	0.006
	NO3-N	0.424	0.005	0.008
	Al	0.414	0.005	0.008
	Ca	0.45	0.002	0.006
