# Supplementary material for: Straw-Mediated Restructure of Arbuscular Mycorrhizal Fungal Community by Selectively Shifting Edaphic Biogeochemistry in Tea Plantations of South Henan, China
Source: J Fungi (Basel). 2026 Apr 9;12(4):271. doi: 10.3390/jof12040271 (PMC13117275; doi:10.3390/jof12040271)
Supplement: Supplementary file 1 [file jof-12-00271-s001.zip › Table S8a-c.pdf]

Table S8-a. The type III tests of fixed effects in the study

Index	Parameters	Numerator degrees of freedom	Denominator degrees of freedom	F	Sig.
MC	Intercept	1	2.94	1427.77	0.000
	Strawtype	1	15.69	1.71	0.210
SD	Intercept	1	2.94	17.31	0.026
	Strawtype	1	19.10	0.05	0.823
Hypha	Intercept	1	2.10	10.29	0.080
	Strawtype	1	19.41	3.17	0.091
GRSP	Intercept	1	2.98	119.23	0.002
	Strawtype	1	19.24	2.40	0.137
Shannon	Intercept	1	2.57	128.19	0.003
	Strawtype	1	19.17	2.07	0.166
Richness	Intercept	1	3.00	22.68	0.018
	Strawtype	1	20.50	0.83	0.372
Pielou	Intercept	1	21.00	633.10	0.000
	Strawtype	1	21.00	0.29	0.595
PD	Intercept	1	3.00	30.94	0.011
	Strawtype	1	20.89	0.51	0.481
PCoA 1	Intercept	1	21.00	0.13	0.721
	Strawtype	1	21.00	0.00	0.957
PCoA 2	Intercept	1	0.85	0.43	0.644
	Strawtype*	1	6.62	5.88	0.048
Positive community	Intercept	1	3.01	32.76	0.010
Cohesion	Strawtype	1	20.91	0.48	0.496
Negative community	Intercept	1	2.95	4.54	0.124
Cohesion	Strawtype	1	20.81	0.08	0.775
Network stability	Intercept	1	9.98	15.21	0.003
	Strawtype	1	49.19	0.01	0.935

Table S8-b The estimates of fixed effects in the study

Index	Parameters	Estimation	Standard Error	Degrees of freedom	t	Sig.
MC	Intercept	57.47	2.23	9.4	25.70	0.000
	Treatment*	-19.34	2.41	21.0	-8.02	0.000
	Strawtype	4.00	3.06	15.7	1.31	0.210
SD	Intercept	131.41	29.20	3.9	4.50	0.011
	Treatment*	-43.18	13.84	18.6	-3.12	0.006
	Strawtype	-4.41	19.41	19.1	-0.23	0.823
Hypha	Intercept	93.99	30.08	6.6	3.09	0.019
	Treatment	-15.34	28.76	20.6	-0.53	0.599
	Strawtype	-67.97	38.16	19.4	-1.78	0.091
GRSP	Intercept	0.37	0.05	8.8	7.15	0.000
	Treatment	-0.06	0.05	20.9	-1.12	0.276
	Strawtype	0.10	0.07	19.2	1.55	0.137
Shannon	Intercept	1.38	0.21	7.9	6.59	0.000
	Treatment	0.26	0.20	20.8	1.27	0.217
	Strawtype	0.39	0.27	19.2	1.44	0.166
Richness	Intercept	13.67	4.31	5.6	3.17	0.021
	Treatment	6.00	3.03	19.5	1.98	0.062
	Strawtype	3.83	4.19	20.5	0.91	0.372
Pielou	Intercept	0.40	0.03	21.0	12.60	0.000
	Treatment	0.00	0.04	21.0	0.00	0.999
	Strawtype	0.02	0.04	21.0	0.54	0.595
PD	Intercept	2.80	0.75	6.4	3.74	0.009
	Treatment	0.89	0.58	19.8	1.53	0.143
	Strawtype	0.57	0.80	20.9	0.72	0.481
PCoA 1	Intercept	-0.08	0.09	21.0	-0.88	0.387
	Treatment	0.17	0.12	21.0	1.47	0.155
	Strawtype	-0.01	0.13	21.0	-0.06	0.957
PCoA 2	Intercept	-0.24	0.07	3.7	-3.57	0.027
	Treatment*	0.37	0.08	20.7	4.83	0.000
	Strawtype*	0.23	0.09	6.6	2.43	0.048
Positive community Cohesion	Intercept	3.59	0.98	6.5	3.67	0.009
	Treatment*	1.62	0.77	19.9	2.11	0.048
	Strawtype	0.73	1.05	20.9	0.69	0.496
Negative community Cohesion	Intercept	-6.30E-05	4.66E-05	7.6	-1.35	0.215
	Treatment	-3.66E-05	4.14E-05	20.4	-0.88	0.387
	Strawtype	1.62E-05	5.58E-05	20.8	0.29	0.775
Network stability	Intercept	1.17E-05	6.50E-06	37.5	1.81	0.078
	Treatment	5.40E-06	7.60E-06	378.9	0.71	0.479
	Strawtype	-7.00E-07	9.10E-06	49.2	-0.08	0.935

Table S8-c. The estimates of covariance parameters about the random effects in the study

Index	Parameters	Estimation	Standard Error	Wald Z	Sig.
MC	Intercept	20.84	6.86	3.04	0.002
	Intercept (Group)	3.66	6.08	0.60	0.547
SD	Intercept	583.94	194.66	3.00	0.003
	Intercept (Group)	2839.20	2425.06	1.17	0.242
Hypha	Intercept	2779.81	946.16	2.94	0.003
	Intercept (Group)	1235.38	1704.27	0.73	0.469
GRSP	Intercept	0.01	0.00	3.03	0.002
	Intercept (Group)	0.00	0.00	0.83	0.408
Shannon	Intercept	0.14	0.05	3.00	0.003
	Intercept (Group)	0.06	0.07	0.78	0.438
Richness	Intercept	28.79	9.56	3.01	0.003
	Intercept (Group)	47.29	42.67	1.11	0.268
Pielou	Intercept	0.01	0.00	3.24	0.001
	Intercept (Group)[a]	-	-	-	-
PD	Intercept	1.07	0.36	3.02	0.003
	Intercept (Group)	1.24	1.17	1.07	0.287
PCoA 1	Intercept	0.05	0.02	3.24	0.001
	Intercept (Group)[a]	-	-	-	-
PCoA 2	Intercept	0.02	0.01	2.64	0.008
	Intercept (Group)	0.00	0.01	0.23	0.821
Positive community Cohesion	Intercept	1.87	0.62	3.02	0.003
	Intercept (Group)	2.10	1.98	1.06	0.288
Negative community Cohesion	Intercept	5.62E-09	1.86E-09	3.02	0.003
	Intercept (Group)	3.70E-09	3.86E-09	0.96	0.337
Network stability	Intercept[a]	2.21E-10	-	-	-
	Intercept (Group)	1.07E-11	2.19E-11	0.49	0.627

Treatment, control (CK) and straw-amended (S) treatment; Strawtype, control (CK), wheat straw (WT), rice straw (RT). a, The parameter is redundant. Test statistics and confidence intervals cannot be computed; *, The parameter is significant in a level of 0.05. Sig., the p-value of the parameter's test statistics.
