# Supplementary figures and images for: Straw-Mediated Restructure of Arbuscular Mycorrhizal Fungal Community by Selectively Shifting Edaphic Biogeochemistry in Tea Plantations of South Henan, China
Source: J Fungi (Basel). 2026 Apr 9;12(4):271. doi: 10.3390/jof12040271 (PMC13117275; doi:10.3390/jof12040271)

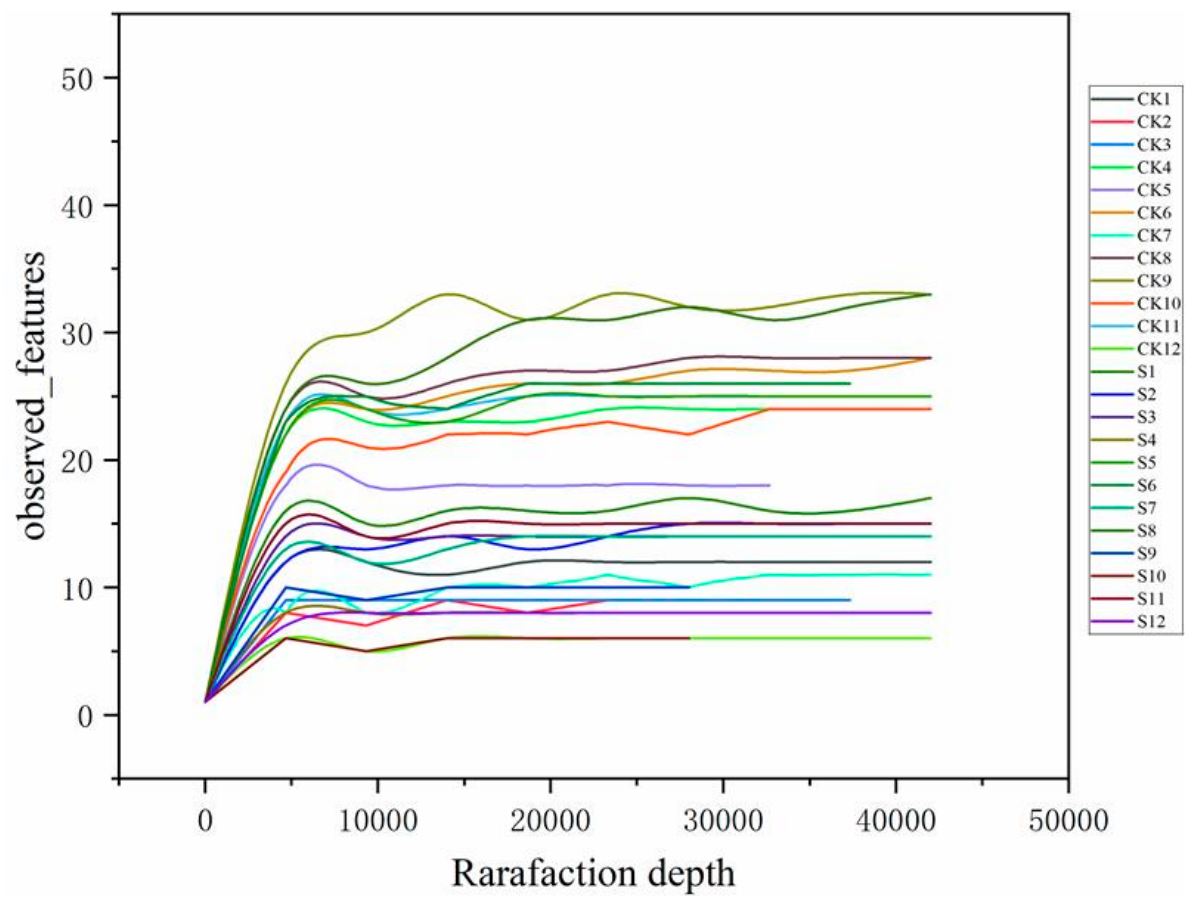

Figure S1. Rarefaction of AM fungi sequencing reads.

Supplement: Supplementary file 1 [file jof-12-00271-s001.zip › Figure S1.pdf]
